# Understanding Mental Health Impact of COVID-19 on Puerto Rican Youth: Influence of Parental Stress

**DOI:** 10.3390/ijerph21121564

**Published:** 2024-11-26

**Authors:** Gabriela M. Martínez-Seda, María C. Vélez-Pastrana, Andel Nicasio-Infante

**Affiliations:** PhD Program in Clinical Psychology, San Juan Campus, Albizu University, San Juan, PR 00901, USA

**Keywords:** COVID-19 pandemic, child mental health, adverse COVID-19 experiences, parental stress, parental psychopathology

## Abstract

The COVID-19 pandemic disrupted all areas of society. The socioemotional effects of isolation, financial instability, and educational and employment uncertainty are anticipated to have a ripple effect on the mental health of parents and children, which has not yet been studied in the Puerto Rican context. To examine the impact of COVID-19 on Puerto Rican families, we used a cross-sectional, correlational research design that studied the following: (a) Adverse experiences (AE) related to the COVID-19 pandemic reported by parents; (b) Parental stress; (c) Parental psychopathology: (d) The mental health of their children. We hypothesized that AEs have direct and indirect effects on child mental health, which are mediated by parental mental health and stress. One hundred and thirty-five parents with children aged 4 to 18 years completed an anonymous online survey from March to May 2022. Results show that COVID-19 AEs have significant direct and indirect effects on child mental health, which are mediated by parental stress and parental psychopathology. We observed medium-to-large effect sizes in the associations between child mental health and COVID-19 AEs, which are mediated by parental stress and psychopathology. Children’s mental health symptoms worsen as parental stress and parental psychopathology increase in the context of COVID-19 AEs.

## 1. Introduction

On 8 March 2020, the first case of COVID-19 was reported in Puerto Rico (PR). The first patient was a European tourist who was visiting the island on board a cruise ship [1]. On 11 March 2020, the World Health Organization (WHO) declared a pandemic caused by the COVID-19 virus. Health officials urged countries to develop a comprehensive government strategy to prevent infections, save lives, and minimize the virus’s impact [2]. Immediately afterward, the government of Puerto Rico declared a state of emergency and issued an executive order mandating a lockdown to mitigate the spread of COVID-19 [3]. Strict social distancing and social isolation measures were enforced in Puerto Rico, with which the public broadly complied.

COVID-19 arrived in March 2020, three years after Hurricane Maria destroyed the electricity grid; currently, power outages still occur regularly, and there is an island-wide shortage of healthcare facilities and workers [9]. The island had not recovered from these events when the COVID-19 pandemic arrived in March 2020, and it was hit hard by the pandemic. Puerto Rico responded with some of the strictest pandemic measures in the U.S., including nonessential-business closures, stay-at-home orders, mask mandates, and high compliance by the people. Cultural, social, and political differences between Puerto Rico and the U.S. have been cited among the factors that promoted the success of COVID-19 vaccination in PR. For example, widespread compliance with strict COVID-19 measures in PR contrasted with the mainland United States where some sectors of the population consistently resisted mask-wearing rules and vaccine mandates, citing “personal liberty” [9]. Remarkably, by October 2021, Puerto Rico had the highest rate of COVID-19 vaccination in the U.S., with more than 73% of the total population fully vaccinated. This surpassed the U.S. national average of just over 57% [9]. The high vaccination rate contrasted with Puerto Rico’s initial increased vulnerability to COVID-19. The first confirmed case was recorded on 9 March 2020. By the end of December, and since 9 March 2020, PR had documented 95,330 confirmed cases and 2085 deaths. The pandemic severely affected the population, which has a high fatality rate among older adults, particularly those over 60 years old [10].

Puerto Rico has a population of around 3.3 million, and almost half of the population of Puerto Rico live below the poverty line, with a poverty rate of 43.5%, according to the 2020 U.S. Census data [4]. This contrasts with the 11.4% poverty rate in the U.S. When the COVID-19 pandemic struck, the island of Puerto Rico had spent more than a decade reeling in the wake of economic, social, and political crises and several consecutive natural disasters. After the 2008 world economic collapse, the U.S. Congress under the Obama Administration imposed an austerity regimen and placed the U.S. territory under the control of a “Junta”, the Fiscal Control Board [5]. Mass migration of Puerto Ricans to the continental U.S. ensued, most of whom were of working and reproductive age [6]. In the period from 2008 to March 2020, Puerto Ricans lived through economic collapse, the debt crisis, and the subsequently imposed austerity; political upheaval and mass popular protests, which forced the governor to resign in 2019 (an interim acting governor ruled during the pandemic); two back-to-back hurricanes in the same week of September 2017—Hurricanes Irma and Maria [7], and a series of earthquakes in early 2020, which destroyed homes and schools [8].

According to a 2023 UCLA report on the impact of COVID-19 on Latinos in the U.S., COVID-19 infection and mortality rates among racial and ethnic minorities are greater than those of white people [11]. Since the pandemic began, minority populations, including Black, Latino, Asian, and Native American people, face higher hospitalization and intensive care unit (ICU) admission rates due to COVID-19, compared to white patients. For example, the hospitalization rate due to COVID-19 for Latinos was 4 times greater than that of white patients, and Latinos’ COVID-19 mortality rate is 9.2 times greater than that of white patients [11]. The 2019 coronavirus disease (COVID-19) outbreak significantly disrupted every area of our lives.

Among the population, children and adolescents have generally exhibited a degree of resistance to the virus, with cases often resulting in milder symptoms and a positive prognosis [12,13]. This led health authorities such as the WHO to recognize that children and adolescents were not as vulnerable compared to older age groups [14]. Despite their physiological resilience, children and adolescents may still be vulnerable to the broader impact of the pandemic and associated mitigation strategies, particularly in terms of their psychological and socioemotional development [15,16]. For this reason, it is crucial to understand the factors that adversely affect child mental health in the context and aftermath of the COVID-19 pandemic in order to identify and deploy protective factors and intervention strategies.

To adequately assess the potential impact of the COVID-19 pandemic on child mental health, it is necessary to understand the factors that have been shown to adversely affect child mental health. Notable among these factors are adverse childhood experiences (ACEs) [17], parental mental health problems or psychopathology [18,19], parental stress [20], setbacks in national economies [21], and family socioeconomic status [19]. To understand their potential impact in the context of the COVID-19 pandemic, we summarize the scientific literature on each of these factors as they relate to child mental health.

### 1.1. Factors That Adversely Affect Child Mental Health

#### 1.1.1. Adverse Childhood Experiences (ACE)

The first factor to consider is Adverse Childhood Experiences (ACEs), which can include abuse, emotional or physical neglect, exposure to natural disasters, medical trauma, the loss of a parent, and the disability of a parent or caregiver due to psychopathology [20]. ACEs are considered a significant factor when evaluating child mental health because ACEs predict psychopathology across lifespans, and they have a strong and persistent association with mental health status in children [17].

#### 1.1.2. Stress and Parental Mental Health

Parental stress is another factor that is highly correlated with child mental health [22,23,24,25]. Abidin defined parental stress as the discrepancy between the situational demand of parenting and the resources for meeting that demand [26]. The stressors experienced by parents can be associated with personal, marital, work, and economic problems, among others [20,27].

Parental mental health is closely associated with parental stress and is also an important predictor of child mental health [28]. The parents’ mental health and their children’s mental health are closely correlated [20]. As psychological stressors, as perceived by the parents, increase, so do their children’s risk of mental illness [20].

#### 1.1.3. Economic Factors

Researchers have shown that the national economic conditions are closely related to child mental health—as a country’s economic conditions worsen, child mental health deteriorates [20]. A study that examined mental health symptoms across five domains in children aged 4 to 17 years showed that the factors that accompany a weakened economy, such as a declining household income and persistent concern about unemployment, are significantly related to worsening child mental health, even after controlling for parental employment [20]. Another study found that the effects of the national economy during the 2008 stock market crash impacted child mental health and had negative effects on children’s emotional and physical well-being [29].

Poor economic conditions are detrimental to child mental health [20]. For example, when a family is affected by a lack of employment, reduced hours and earnings, and the pressure to meet the debt obligations of the parents, this increases social and psychological stress, which in turn are detrimental to the mental health of their children. Furthermore, lack of money and resources in the family may mean that the children may not have access to the mental health services that they need in order to develop and maintain adequate mental health [20]. Lupien and colleagues found that the psychological effects extend to physiological responses. Specifically, they report that children with low socioeconomic status present significantly higher salivary cortisol levels than children with high socioeconomic status; this difference is evident as early as age 6 [30].

### 1.2. The COVID-19 Pandemic and Child Mental Health

In the field of mental health, experts have highlighted how the pandemic has exacerbated and heightened the factors that had already been shown to have a detrimental effect on child mental health [31,32]. The COVID-19 pandemic affected the global economy, which had a direct effect on families’ socioeconomic status [32]. Families found themselves with more stress associated with the added pressures, new responsibilities, and the loss of support systems because of the social isolation and quarantine that resulted from the pandemic [31,32,33,34].

**Figure 1:**
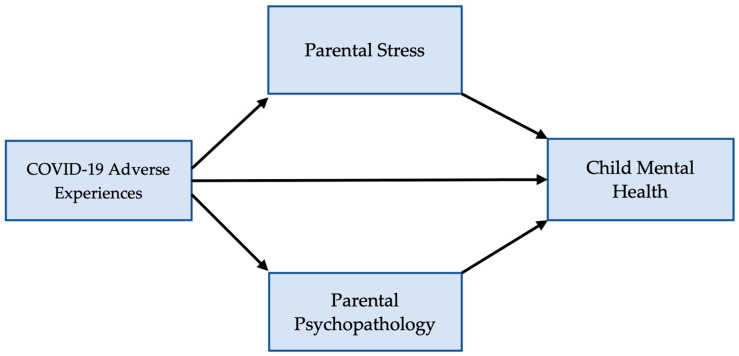
Proposed Parallel Multiple Mediator Model of COVID-19 experiences as a predictor of child mental health, which are mediated by parental stress and parental psychopathology.

To improve our understanding of the pandemic’s impact on children’s well-being, it is important to examine the multiple intertwined familial factors that provide the context to child development, as well as the exacerbation of these by the pandemic and the additional stressors it entailed. Importantly, the specific mechanisms through which the adverse events (AEs) associated with the COVID-19 pandemic affect children’s mental health are not well understood [15]. Addressing this gap requires examining both the direct and indirect ways in which the COVID-19 pandemic may adversely impact child mental health. To this end, this study examined the associations of adverse COVID-19 experiences (i.e., COVID-19 AE), parental stress, parental psychopathology, and child mental health in terms of direct and indirect (mediated) effects. We tested a parallel multiple mediator model of adverse COVID-19 experiences as a predictor of child mental health, which are mediated by both parental stress and parental psychopathology, in order to examine the potential mediating role of parental stress and parental psychopathology in the relationship between COVID-19 AE and child mental health. This model is presented in Figure 1.

## 2. Materials and Methods

### 2.1. Participants

Participants were adults 21 years or older who are the parent, legal guardian, or caregiver of a child between the ages of 4 and 18 years and had resided in Puerto Rico since March 2020, specifically during the time of the COVID-19 Pandemic. A convenience sample of 309 adults was recruited through social media and institutional mailing lists. These participants responded to an anonymous online survey, which consisted of self- and other-report questionnaires that measured the variables of this study. Participation was voluntary, and informed consent was obtained from all participants. The entire questionnaire was answered online using the Survey Monkey platform, and no identifying data were collected (e.g., name, IP 280 address). If families consisted of more than one child, parents were asked to choose the child whose upbringing had represented the greatest challenge during the COVID-19 season to reduce sources of error, ensure uniformity and reduce bias.

### 2.2. Measures

#### 2.2.1. Child Mental Health

The Strengths and Difficulties Questionnaire (SDQ) was used to measure child mental health, which is the criterion variable of this study. The instrument consists of 25 items that present different symptoms and problems, such as “often complains about headaches”, “often has temper tantrums”, and “is restless, overactive…”. Respondents indicate the extent to which each symptom applies to their child, using a three-point Likert-type scale, with the options “Not True”, “Somewhat Truec, or “Certainly True”, scored 0, 1, or 2. The SDQ refers to a time frame of the previous 6 months, or “during the present school year”. It can be completed by parents and teachers of children aged 4–16 and by adolescents aged 11–16 in under five minutes [35]. Its 25 items are organized into the following five subscales: Emotional Problems, Conduct Problems, Hyperactivity, Peer Problems, and Prosocial behavior. The scores for each subscale can range from 0 to 10 if all the items are completed [36]. Emotional problems, conduct problems, hyperactivity, and peer problems subscales are added together to generate a Total Difficulties score that ranges from 0 to 40. Higher scores indicate more symptoms or “worse” mental health in the child. The parent-report form of the SDQ has good internal consistency (α = 0.77) [37]. In the present sample, the Total Difficulties scale had an internal consistency α = 0.85. The different subscales had the following internal consistency: Emotional problems 0.79, Conduct problems 0.64, Hyperactivity 0.76, and Peer problems 0.67.

#### 2.2.2. Parental Psychopathology

The Parental Mental Health Status Kessler 10 (K10) [38] was used to measure parental psychopathology, one of the mediator variables in the study. The K10 is a brief screening tool comprising 10 items that ask how frequently the respondent has experienced symptoms of psychological distress related to anxiety and depression during the past four weeks. For example, “In the last four weeks, how often have you felt tired out for no good reason?”. Responses are recorded on a 5-point Likert-type ordinal scale ranging from 1 (“Always”) to 5 (“Never”). It takes approximately 5 min to complete. The K10 is scored by adding the 10 items. Possible scores range from 10 through 50, and higher scores represent high levels of psychological distress. Cut-off scores for the K10 are low (10–15), moderate (16–21), high (22–29) and very high (30–50). The K10 Spanish version has shown an internal consistency of 0.90 [39]. In the present sample, the K10 had an internal consistency α = 0.94.

#### 2.2.3. Parental Stress

The Perceived Stress Scale (PSS) was used to measure parental stress, a mediator variable in our model. The Perceived Stress Scale was developed by Cohen, Jamarck, and Ermelstein to measure the degree to which a person perceives situations in their life as stressful [40]. The scale consists of 14 items, which are answered according to the degree to which the individual perceives life as unpredictable, uncontrolled, or overwhelming in a time frame of eight weeks. The items are answered on a 5-point Likert-type scale ranging from Never (0) to Very Often (5). Possible scores range from 0 to 56; higher scores indicate greater self-reported parental stress. Remor reports that the Spanish version of the PSS has adequate internal and test-retest reliability (α = 0.81; r = 0.73, respectively) [41]. Furthermore, they report that the PSS has good sensitivity in detecting populations under different stress levels. They also report good concurrent validity, with positive correlations between the PSS and measures of similar constructs, such as distress and anxiety. The PSS had an internal consistency index of α = 0.88 in the present study.

#### 2.2.4. Adverse COVID-19 Experiences

The researchers developed a checklist/questionnaire to assess the participant’s exposure to adverse experiences during the period of the COVID-19 pandemic of 2020–21. The items were developed according to a review of the literature and current events related to the evolving COVID-19 situation. This study was developed and submitted for IRB approval as the pandemic was evolving, and the project and questionnaire evolved according to the changing situation in PR (i.e., full lockdown ended, vaccination became available, in-person activities slowly reinitiated, etc.). This enabled the AE questions to better reflect the changing reality and not be rendered obsolete by the time data were collected. For example, we added questions about AE’s related to vaccination access and refusal as vaccines became available during the conceptualization and development phase of the project. Up until IRB approval for data collection in late 2021, the questionnaire was continuously revised to reflect changing circumstances. Therefore, we were able to include COVID-19-AE questions that reflect adverse experiences, which ranged from the early pandemic stressors (the deaths and sickness, loss of loved ones, of jobs and income, the strict lockdowns), through the advent of vaccines and the gradual easing of some restrictions, but that also introduced new stressors—related to vaccine hesitancy, stigma, related conflicts at the workplace or school, and also the very long and enduring school closures in PR and ensuing social isolation, reduced opportunities for social participation, etc.).

The questionnaire consists of 45 binary (yes/no) items scored 1/0, describing adverse experiences or situations to which the participant may have been exposed during the pandemic period (March 2020 to present). They were also asked to select one of these experiences that had been the most salient during the past 4-week period. They were then asked to rate their stress level associated with that experience using a scale of 0 to 10. The situations are organized into seven different categories, which include work, economic difficulties, family stressors, health, death, and loss issues, education, and social support. Some examples are as follows: “I lost my job due to the COVID-19 pandemic” and “A family member got sick with COVID-19”.

The COVID-19 AE scores were computed from the sum of the 45 items that referred to all adverse experiences or situations experienced during the pandemic period (March 2020 to present). The checklist demonstrated good internal consistency (α = 0.78).

### 2.3. Data Analyses

This observational study had a cross-sectional, correlational research design. First, we conducted descriptive analyses of all study variables, using means and standard deviations and absolute and relative frequencies for continuous and categorical variables, respectively. Next, we examined the associations of the predictor variable (adverse COVID-19 experiences checklist) and mediator variables (PSS and K10 scores for parental stress and parental psychopathology, respectively), as well as the outcome variable (SDQ scores for child mental health) and variables among themselves. For this, we used Pearson’s r and Chi-Square tests for continuous and categorical variables, respectively.

Lastly, we tested the hypothesized parallel multiple mediator model of COVID-19 experiences as a predictor of child mental health, which is mediated by parental stress and parental psychopathology. We conducted our mediation analyses using the PROCESS Procedure for SPSS Version 4.1, developed by Hayes [42]. We used model 4 “Parallel Multiple Mediator Model”, which allowed us to estimate the direct and indirect pathways from the predictor, adverse COVID-19 experience scores, to the outcome, SDQ scores, using PSS scores and K10 scores as the two mediators. As Hayes explains, in parallel multiple mediator models, the antecedent variable (COVID-19 AE) is modeled as influencing the consequent variable (SDQ scores) directly and also indirectly through two or more mediators (PSS and K10 scores). A parallel multiple mediator model with 2 mediators like ours has 3 consequent variables, and so requires three regression equations to estimate all the effects of COVID-19 AE on SDQ scores. In this set of equations, *a1* and *a2* estimate the effects of COVID-19 AE on PSS scores and K-10 scores, respectively; in turn, *b1* and *b2* estimate the effects of PSS scores and K-10 scores on SQD scores, controlling for both COVID-19 AE and the other mediator (K-10 or PSS scores). Finally, *c′* estimates the effect of COVID-19 AE on SDQ scores, holding the two mediators (PSS and K-10 scores) constant. As Hayes explains, estimating indirect effects on a parallel multiple mediator model like ours allows one to simultaneously test for each mechanism while accounting for the association between them. We used SPSS version 25 to conduct all other analyses.

## 3. Results

### 3.1. Participants’ Characteristics

Participants in this study were 309 parents who provided information about themselves and their children; these parents completed all the study questionnaires. The adult participants’ demographic characteristics are presented in Table 1. The demographic characteristics of the target child about which they report are presented in Table 2. In terms of ethnicity, all participants were Latino/Hispanic, Spanish-speaking Puerto Ricans residing in Puerto Rico. Most of the parents identified as female (95.1%, or 294 of 309); only 15 participants identified as male. The parents’ age range was 22 to 64, with a mean age of 40.79 (SD = 7.59), and the majority was married or cohabitating (227, or 73.5%), employed (251, or 81.2%), and had a college degree (249, or 80.6%). In terms of family composition and size, the median number of people living in the household was 4 (SD = 0.95), and the median number of children living in the household was 2 (SD = 0.87).

Approximately half of the children (161 of 309, or 52.1%) about which the responding parent reported were male (the child’s gender was missing in one case). The child’s age ranged from 4 to 18, with a mean of 10.98 (SD = 4.19) years. Most of the participating parents reported that they had been vaccinated for COVID-19 (295, or 95.5%), and most (268, or 86.7%) also reported that their target child had been vaccinated.

### 3.2. Correlations Among Study Variables

The first objective of the study was to examine the associations between adverse COVID-19 experiences, parental stress, parental psychopathology, and child mental health—the predictor, mediator, and outcome (criterion) variables, respectively. Accordingly, we first examined the correlations among all variables. These results are presented in Table 3. Table 3 presents zero-order Pearson correlations between scores on the COVID-19 AE, PSS, K-10, and SDQ measures. SDQ scores, the criterion variable, had significant, direct correlations of medium effect sizes with the predictor variable, COVID-19 AE, and with both mediators, K-10 and PSS scores. These are presented in Table 3 and range in magnitude from 0.385 to 0.365 (*p* < 0.01). COVID-19 AE scores were positively correlated with both mediators, K-10 and PSS scores. These correlations were significant and of moderate effect sizes (0.365 to 0.291, *p* < 0.01). Finally, the two mediators, the PSS and K-10 scores, had a significant direct correlation with a large effect size (0.761, *p* < 0.01).

In summary, we observed medium-to-large, significant, and positive correlations between SDQ, PSS, K-10, and COVID-19 AE scores. This means that children’s mental health symptoms (SDQ scores) tend to increase as parental stress (PSS) scores and parental psychopathology (K-10) scores increase. Also, familial exposure to a greater number of COVID-19 AEs is significantly associated with higher parental stress (PSS), greater parental psychopathology (K-10), and reduced child mental health (as indicated by higher SDQ scores).

### 3.3. Mediation Analyses

**Figure 2:**
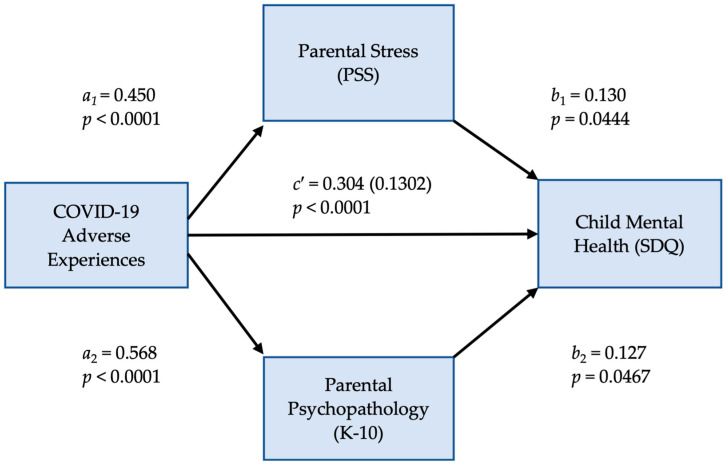
Parallel Multiple Mediator Model of COVID-19 experiences as a predictor of child mental health, which are mediated by parental stress and parental psychopathology.

The second objective of the study was to evaluate the hypothesized Parallel Multiple Mediator Model. We used COVID-19 AE as the predictor variable, PSS scores, and K-10 scores as mediators, and SDQ scores as the outcome (criterion) variable. These results are presented in Figure 2 and Table 4. The parallel multiple mediator model with two mediators that we tested to estimate all the effects of COVID-19 AE on SDQ scores has three consequent variables and requires three regression equations (Hayes, 2022). The results from these regressions are in Figure 2 and Table 4.

Figure 2 depicts the statistical diagram of the model that we tested. As presented in Figure 2, we observed a significant direct effect of COVID-19 AE on SDQ scores (*c′* = 0.304, SE = 0.069, *p* < 0.0001). There was also a significant indirect effect of COVID-19 AE on SDQ scores (0.1302) that was partially mediated by PSS scores (*a*_1_*b*_1_ = 0.0583) and K-10 scores (*a*_2_*b*_2_ = 0.0719). That is, adverse COVID-19 experiences affect child mental health (SDQ) directly and indirectly, as their effect is mediated by the effect that COVID-19 AEs have on parental psychopathology (K-10) and parental stress (PSS).

Table 4 presents the coefficients in the three equations. As presented in Table 4, while COVID-19 AE explains 9% of the variance of PSS scores (R^2^ = 0.085) and 13% of the variance of K-10 scores (R^2^ = 0.126), about 21% of the variance in SDQ scores is significantly explained by both mediators, PSS and K-10 scores, together with COVID-19 AE (R^2^ = 0.214).

## 4. Discussion

The present study examined how the adverse experiences that many families lived through during the COVID-19 pandemic may impact children’s mental health directly and indirectly through their effect on parental stress and parental psychopathology. This serves the overarching goal of understanding the effects that the COVID-19 pandemic has exerted on children’s mental health and the mechanisms through which these negative effects are conveyed.

### 4.1. Relationships Between Variables

As hypothesized, the results of our preliminary correlation analyses show medium-to-large, significant, and direct correlations between children’s mental health symptoms, parental stress, parental mental health, and adverse COVID-19 experiences. Thus, our results suggest that children’s mental health symptoms intensify as their parents’ stress levels increase. Children’s mental health symptoms are also intensified in families where the parents have more psychopathology symptoms. This is consistent with the research that shows that parental stress is significantly associated with children’s psychological symptoms [43] and that higher levels of parental stress are related to children’s mood and anxiety problems [27,44]. Other researchers have suggested that the relationship between parental stress and child symptoms could be explained by a negative family environment, which is created by parental stress. This environment, in turn, increases the probability that children will develop internalizing and externalizing symptoms [45]. This is especially relevant in the context of the COVID-19 pandemic, considering that the pandemic presented a chronic stressor that could potentially lead to depleted resources and serious long-term effects [46].

Furthermore, we found that as the number of adverse COVID-19 experiences to which the family is exposed increases, parental stress levels and parental psychopathology symptoms also increase. The association between adverse experiences and parental stress levels is consistent with research prior to the COVID-19 pandemic [47]. Rose and colleagues examined the impact of economic hardship, caregiver emotional distress, partner conflict, and adverse childhood experiences on children’s cognitive, behavioral, and social outcomes. This stress increased as economic and other pressures increased [47], and greater stress led to negative child development outcomes. Rose et al. did not identify a pathway that related familial pressure to ACES. This discrepancy between our findings may be because the adverse COVID-19 experiences identified a wider range of scenarios that were typical in the context of the pandemic and are not limited to the typical definition of ACES.

Our results show that in the context of the COVID-19 pandemic and the associated difficulties, greater COVID-related adverse experiences correlated with an increase in parental stress. Similarly, Jarvers and colleagues have shown that parental stress increases as preventative lockdown measures are put in place, which results in additional burdens for parents [44]. Increased parental stress during the COVID-19 pandemic is associated with changes in children’s daily structures and routines, worry and anxiety around COVID-19, and demands related to children’s online schooling [48]. Parental stress emerges as an important variable for intervention to minimize the negative effects of the pandemic on children [49].

Our findings also indicate that parental psychopathology was also adversely affected in the context of increasing adverse COVID-19 experiences. This is consistent with previous research [49,50]. Economic hardship, anxiety, loneliness, stigma, and increased substance use due to COVID-19 have been previously associated with the decline of parental mental health in the context of COVID-19 experiences [51].

We found that child mental health deteriorates with increases in the number of adverse COVID-19 experiences to which their family is exposed; children whose families are exposed to more adverse experiences across diverse categories have more mental health symptoms. This dose-dependent relationship is consistent with studies, which show that COVID-19 stress is significantly associated with child mental health symptoms, particularly externalizing symptoms [48,50,52]. This is consistent with pre-pandemic research that shows an association between increased familial stressors and exacerbated mental health symptoms in children and adolescents [53,54].

We examined the role of COVID-19 experiences as a predictor of child mental health that has direct and indirect effects; we proposed that these indirect effects are mediated by parental stress and by parental psychopathology. We tested a parallel multiple mediator model to examine the mediating role of parental stress and parental psychopathology in the relationship between adverse COVID-19 experiences and child mental health (see Figure 1).

### 4.2. Mediated Effects

Our results show that the adverse experiences that families lived through during the COVID-19 pandemic have significant direct and indirect effects on child mental health, consistent with our hypothesized model (Figure 1). Children whose families are exposed to more COVID-19 adversities exhibit more mental health symptoms, and adverse COVID-19 experiences affect child mental health both directly and indirectly. That is, the effects that the family’s exposure to adverse COVID-19 experiences have on child mental health were partially mediated by parental stress and parental psychopathology. Increased exposure to a variety of adverse events related to the COVID-19 pandemic (such as job loss, illness, financial hardship, loss of support networks, etc.) was associated with increased parental psychopathology and increased parental stress. The effect of COVID-19-related adversities is transmitted indirectly via the impact that adversities have on both parental stress and parental psychopathology. Exposure to more adverse COVID-19 experiences increases parental stress, and increased parental stress, in turn, exacerbates child mental health symptoms. The pandemic seemed to have an amplified effect on child mental health by disrupting the family system.

The mediating role of parental stress has been previously identified in existing literature [55]. The concept of “stress contagion” as proposed by Liu and Doan can help interpret the mediating role of parental stress to transmit the effects of adverse COVID-19 experiences and thus worsen children’s mental health [56]. “Stress contagion” refers to the direct and indirect effects of pandemic-related stress that may be exacerbated and multiplied due to a process of stress proliferation among members. Stress contagion can be classified in two ways: spillover, in which the stress in one domain impairs one’s ability to function in another, and crossover, in which stress in one member of the family leads to increased stress for another member [56]. In our study, both types of stress contagion were consistent with the adverse experiences identified. The parent’s stress was increased by work demands and economic difficulties. The increase in stress may, in turn, have impaired their ability to effectively function in the parenting domain, which was now demanding more from parents than before. This bidirectional relationship among the adverse experiences explains the pandemic’s effect on children’s mental health and overall family well-being.

Consistent with existing literature, our findings also show that exposure to more adverse COVID-19 experiences exacerbates parents’ psychopathology symptoms, which, in turn, negatively affects their children’s mental health [56,57]. It has been proposed that the mediating effect of parental psychopathology can be understood through the cyclical challenge parents face in caring for their children and the disruptions caused by their mental health conditions [58]. This effect is heightened when compounded by socioeconomic challenges and alleviated when the family has a robust support system, both of which were impacted by adverse COVID-19 experiences [16]. In our study, increased adverse COVID-19 experiences were related to greater anxiety and depressive symptoms in parents. This is consistent with research by Brown and colleagues that showed that greater COVID-19-related stressors and high anxiety and depressive symptoms are associated with higher parental perceived stress [57]. On the other hand, previous research also highlights the role of parental self-regulation in mitigating the adverse effects of parental psychopathology on child mental health, an ability that may be compromised when psychological symptoms such as anxiety and depression occur [57].

### 4.3. Implications

The findings of this study, which examines the relationships between adverse COVID-19 experiences, parental stress, parental psychopathology, and child mental health, have significant implications for public policy and practice. These implications are particularly relevant given the importance of addressing the potential long-term consequences of the pandemic. Interventions targeting parental stress and psychopathology are crucial for mitigating the adverse effects of the COVID-19 pandemic on child mental health. This may include providing support services to cope with stressors and access to mental health resources. On the other hand, it is important to consider the role of parenting skills in buffering the direct and indirect effects of adverse COVID-19 experiences. Anticipating the possible effect of COVID-19’s disruption in the family system, Acevedo-Velázquez and Vélez Pastrana suggest various parent-training psychoeducational interventions. These could be applied virtually to develop parenting skills, which may increase parental competence and also promote parental and child well-being [59].

Further research is warranted to better understand the complex pathways between the COVID-19 pandemic and child health outcomes. Longitudinal studies may provide insight into the temporal relationships and causal pathways involved. It is also necessary to explore the cultural and contextual factors that may influence the impact of COVID-19 on family systems and mental health outcomes to develop and improve culturally sensitive interventions. Based on such research, it is pertinent to study the effectiveness of interventions targeting parental stress and psychopathology and their indirect impacts on child mental health in order to inform evidence-based practice. Finally, long-term follow-up studies may help us understand the enduring effects of the pandemic on the family system and on children’s mental health.

### 4.4. Strengths and Limitations

The present study examined the effect of adverse COVID-19 experiences on children’s mental health, considering both direct and indirect pathways through parental stress and psychopathology in a sample of Spanish-speaking Puerto Rican parents residing in Puerto Rico. A significant strength of our study is that it focuses on an underserved ethnic minority population. Thus, it adds value to what may be an established body of scientific research by focusing on populations that are grossly underrepresented in such research. It is a worthwhile endeavor to examine how what we know about the associations among parental stress, adverse external events, and child mental health may vary across ethnicities and cultures. This knowledge is based on the existing scientific literature, which tends to focus on and disproportionately represent different kinds of peoples and populations [60,61,62]. We do know that there are racialized, cultural, and ethnic differences in health and its social determinants.

Furthermore, ethnic and cultural differences may impact the salience of adverse experiences, perceived stress, and parental and child mental health, the variables that we studied. For example, “Familism” is a core value in Puerto Rican culture, where family ties, intergenerational bonds, and loyalty are prioritized [63]. This may mean that measures taken during the pandemic, including lockdowns, social distancing, and being isolated from extended family, could have differential effects in the Puerto Rican context compared to other ethnic groups who emphasize individualism and have more nuclear family dynamics. Similarly, cultural and socioeconomic factors may upset the associations among the variables that we study. For example, although in Puerto Rican culture, traditional gender roles are valued, prioritizing the father’s role as the main breadwinner, many households are single-parent families, predominantly headed by single mothers [63]. This situation has obvious implications in the context of the COVID-19 pandemic and lockdowns. Social isolation, having multiple roles, economic hardship, and lack of support may have differentially shaped the experiences and perceptions of Puerto Rican parents and their children. On the bright side, the cultural and political differences between Puerto Rico and the U.S. have been cited among the factors that promoted the success of COVID-19 vaccination in PR. The Puerto Ricans’ emphasis on family and community, as opposed to the U.S. culture of individualism, may have influenced the widespread compliance with strict COVID-19 measures that occurred in PR.

Although the convenience sampling strategy enabled us to gather a sample of 309 participants, the inherent limitation of this recruitment method is that it may not fully represent the target population, thereby restricting the ability to generalize the findings. For example, female parents were overrepresented. Another limitation is that the study focuses on the parent’s perspective. Future studies should include parents of different genders, other age groups, children with disabilities, and other circumstances that may represent an added layer of difficulties for parents. Additionally, the study’s cross-sectional design limits any inferences about causality or temporal sequence. Longitudinal studies may be key in providing the necessary evidence to determine those relationships. However, the nature of the circumstances contextualizing the study, i.e., the COVID-19 pandemic, would have been difficult to anticipate as required for a longitudinal design.

Another significant strength of this study is its relevance to the current context. The study addressed a timely topic and focused on a population that is underrepresented in research. This project was developed and submitted for IRB approval as the pandemic was evolving, and the project and questionnaire evolved according to the changing situation in PR (i.e., full lockdown ended, vaccination became available, in-person activities slowly reinitiated, etc.). This enabled the AE questions to better reflect the changing reality and not be rendered obsolete by the time data were collected. For example, we added questions about AE’s related to vaccination access and refusal as vaccines became available during the conceptualization and development phase of the project.

## 5. Conclusions

This study sheds light on the complex dynamics between adverse COVID-19 experiences, parental stress, parental psychopathology, and child mental health. In conclusion, there were direct and indirect effects of adverse COVID-19 experiences on child mental health, which were mediated through parental stress and parental psychopathology. These results underscore the importance of addressing the family system, parental well-being, and family dynamics in interventions aimed at promoting children’s mental health resilience during challenging times such as the COVID-19 pandemic. Addressing the mental health needs of children and families affected by the COVID-19 pandemic requires a multifaceted approach that considers the interplay of individual, systemic, and societal factors. Moving forward, targeted efforts to support parents, develop coping mechanisms, and strengthen social support networks are essential for mitigating the adverse effects of the pandemic on children’s mental well-being.

## Figures and Tables

**Table 1:** Sociodemographic Characteristics of Puerto Rican Parents of children aged 4–18 years (N = 309 ^a^).

Characteristic	Total Sample
	*f* (%)
Age M (Standard Deviation)	40.79 (7.59)
Gender	
Female	294 (95.1)
Male	15 (4.9)
Marital Status	
Single	82 (26.5)
Married or cohabitating	227 (73.5)
Education	
College degree	249 (80.6)
Associate/technical degree or less	60 (19.4)
Employment	
Employed	251 (81.2)
Unemployed or retired	58 (18.8)
Income < 50 K/year in USD	171 (55.3)
Number of people in household Mdn (SD)	4 (0.95)
Number of children in household Mdn (SD)	2 (0.87)
COVID-19—vaccinated	295 (95.5)

^a^ Sample size varies due to missingness.

**Table 2:** Sociodemographic Characteristics of Children, as reported by Puerto Rican parents of children aged 4–18 years in the sample (N = 309 ^a^).

Characteristic	Total Sample
	*f* (%)
Age M (Standard Deviation)	10.98 (4.19)
Gender	
Female	147 (47.6)
Male	161 (52.1)
Type of schooling/education	
Private school	201 (65.0)
Public school	84 (27.2)
Home schooled/other/not in school	24 (7.8)
Child’s grade	
Not in school	4 (1.3)
Nursery/preschool	40 (12.9)
Elementary	108 (35.0)
Middle school	68 (22.0)
High school	80 (25.9)
College	9 (2.9)
Child is vaccinated—COVID-19	268 (86.7)

^a^ Sample size varies due to missingness.

**Table 3:** Descriptive statistics and zero-order correlations for study variables: Measures of adverse COVID-19 experiences, parental stress, parental psychopathology, and child mental health for Latino parents of children ages 4–18 years (N = 309 ^a^).

Variable	*n*	*M*	*SD*	1	2	3	4
1. SDQ	309	12.46	6.91	__			
2. K-10	291	22.97	9.19	0.385 **	__		
3. PSS	283	22.40	8.90	0.365 **	0.761 **	__	
4. COVID-19 AE	309	16.08	5.83	0.369 **	0.365 **	0.291 **	__

Note. SDQ = Strengths and Difficulties Questionnaire total score (child mental health); K-10 = Kessler Psychological Distress Scale (parental psychopathology); PSS = Perceived Stress Scale (parental stress); COVID-19 AE = Adverse COVID-19 Experiences Questionnaire. ^a^ Sample size varies due to missingness; pairwise deletion was used to handle missing data. ** *p* < 0.01.

**Table 4:** Regression coefficients, standard errors, and model summary information for the Parallel Multiple Mediator Model of COVID-19 experiences as a predictor of child mental health, which are mediated by parental stress and parental psychopathology.

Antecedent		Consequent										
		(*M*_1_) PSS				(*M*_2_) K-10				(*Y*) SDQ Scores		
		Coeff.	*SE*	*p*		Coeff.	*SE*	*p*		Coeff.	*SE*	*p*
COVID-19 AE	*a*_1_	0.450	0.088	<0.0001	*a*_2_	0.568	0.089	<0.0001	*c′*	0.304	0.069	<0.0001
*M*_1_ PSS		-	-	-		-	-	-	*b*_1_	0.130	0.064	0.0444
*M*_2_ K-10		-	-	-		-	-	-	*b*_2_	0.127	0.063	0.0467
Constant	*i_M_*_1_	15.182	1.505	<0.0001	*i*_M2_	13.978	1.524	<0.0001	*i_Y_*	1.751	1.292	0.1764
		*R*^2^ = 0.085				*R*^2^ = 0.126				*R*^2^ = 0.214		
		*F*(1, 281) = 25.99, *p* < 0.0001				*F*(1, 281) = 40.36, *p* < 0.0001				*F*(3, 279) = 25.33, *p* < 0.0001		

Note. SDQ = Strengths and Difficulties Questionnaire total score (child mental health); K-10 = Kessler Psychological Distress Scale (parental psychopathology); PSS = Perceived Stress Scale (parental stress); COVID-19 AE = Adverse COVID-19 Experiences Questionnaire; *a*_1_ and *a*_2_, paths from the independent variable to mediators; *b*_1_ and *b*_2_, paths from mediators to the dependent variable; *c′*, direct effect of the independent variable on the dependent variable; *iM*_1_ and *iM*_2_, indirect effects via mediators *M*_1_ and *M*_2_.

## Data Availability

The raw data supporting the conclusions of this article will be made available by the authors on request.
